# Single-Mode Emission by Phase-Delayed Coupling Between Nanolasers

**DOI:** 10.1021/acsphotonics.4c01230

**Published:** 2025-05-05

**Authors:** T. V. Raziman, Anna Fischer, Riccardo Nori, Anthony Chan, Wai Kit Ng, Dhruv Saxena, Ortwin Hess, Korneel Molkens, Ivo Tanghe, Pieter Geiregat, Dries Van Thourhout, Mauricio Barahona, Riccardo Sapienza

**Affiliations:** † Blackett Laboratory, Department of Physics, 4615 Imperial College London, London SW7 2AZ, U.K.; ‡ Department of Mathematics, Imperial College London, London SW7 2AZ, U.K.; § 54174 IBM Research Europe—Zürich, Rüschlikon, Zürich 8803, Switzerland; ∥ School of Physics and CRANN Institute, 8809 Trinity College Dublin, Dublin 2, D02 PN40, Ireland; ⊥ Photonics Research Group, 26656 Ghent University - Imec, Gent 9052, Belgium; # Physics and Chemistry of Nanostructures Group, Department of Chemistry, Ghent University, Gent 9000, Belgium; ∇ Center for Nano—and Biophotonics, Ghent University, Gent 9052, Belgium

**Keywords:** single-mode laser, nanolaser, nonlinear dynamics, parity-time symmetry, exceptional point

## Abstract

Near-field coupling between nanolasers enables collective high-power lasing but leads to complex spectral reshaping and multimode operation, limiting the emission brightness, spatial coherence, and temporal stability. Many lasing architectures have been proposed to circumvent this limitation based on symmetries, topology, or interference. We show that a much simpler and robust method exploiting phase-delayed coupling, where light exchanged by the lasers carries a phase, can enable stable single-mode operation. Phase-delayed coupling changes the modal amplification: for pump powers close to the anyonic parity-time (PT) symmetric exceptional point, a high phase delay completely separates the mode thresholds, leading to single-mode operation. This is shown by stability analysis with nonlinear coupled mode theory and stochastic differential equations for two coupled nanolasers and confirmed by a realistic semianalytical treatment of a dimer of lasing nanospheres. Finally, we extend the mode control to large arrays of nanolasers featuring lowered thresholds and higher power. Our work promises a novel solution to engineer bright and stable single-mode lasing from nanolaser arrays with important applications in photonic chips for communication and LIDAR.

## Graphical abstract

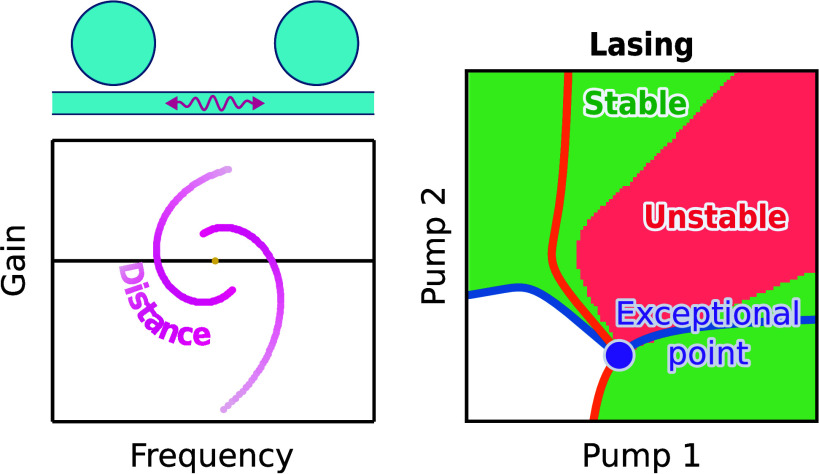

## Introduction

Integrated nanolasers find applications in diverse fields including optical communication,^1,2^ on-chip computing,^3–5^ and LIDAR.^5–8^ Many of these applications, however, require substantial output power in a stable single mode. Whereas subwavelength nanolasers allow stable single-mode operation but are limited in gain, larger high-power nanolasers support multiple spatial and spectral modes, resulting in fluctuations in emission wavelength and power.^9–11^

Coupling many single-mode nanolasers is not a solution to increase the output power, stability, or functionalities, as it leads to complex spectral reshaping and multimode operation, which limits the emission brightness, spatial coherence, and temporal stability. Approaches to suppress additional modes in large and collective nanolasers have explored topology, e.g., periodic nanostructures to create photonic crystals and topological lasers,^12–14^ symmetry,^15^ interference between bright and dark modes, as for bound states in the continuum,^16,17^ and geometrical perturbations.^18–20^ However, these methods require high nanofabrication accuracy, which hinders practical applications.

Non-Hermitian interaction between coupled lasers can be used to achieve single-mode lasing by operating near the exceptional point (EP) where parity-time (PT) symmetry is broken.^21–26^ Although coalescing of eigenmodes at the EP prevents multimode operation, practical realization is limited by its extreme sensitivity to the unavoidable inhomogeneities in realistic systems, often exploited for sensing.^27,28^ Achieving EPs with more than two nanolasers is still an open challenge.^29,30^ A generalized anyonic PT symmetry can be retrieved through complex non-Hermitian coupling between the lasers, resulting in an anyonic EP.^31–34^ However, strong geometric constraints remain since the coupling needs to match the frequency detuning exactly.^31^ When the non-Hermitian coupling becomes purely imaginary (dissipative), coupled lasers can be made to synchronize where their frequencies are locked to each other.^35,36^

Here, we use phase-delayed coupling, where light exchanged by the lasers carries a phase,^37^ to achieve single-mode lasing in large arrays of coupled nanolasers. We employ nonlinear coupled mode theory (CMT) and stability analysis of two coupled nanolasers to demonstrate that, for a high enough phase delay, the modes have different gains and a unique stable lasing mode exists. We link the transition from multimode to single-mode behavior to the appearance of anyonic PT symmetry, but we operate far from the EP, removing stringent restrictions on parameters to achieve mode degeneracy. We further confirm the single-mode operation with a semianalytic treatment of two coupled nanospheres. Finally, we generalize our result to larger arrays, comprising 10 lasers, which also sustain a single mode. Our results promise a novel direction toward low-complexity fabrication of integrated nanolasers with stable, high-power single-mode operation.

## Results and Discussion

### Single-Mode Lasing From a Phase-Delayed Dimer

We first show that a system of two frequency-detuned coupled resonators can achieve single-mode lasing by increasing the phase delay of the coupling between them, using linear CMT and a coupling element *κe* ^*i*ϕ^, where ϕ is the phase delay.

Under real coupling (ϕ = 0, Figure 1a), the modes repel in real frequency [Re­(ω)], when the lasers are pumped equally. The threshold pump required for each mode to initiate lasing, -Im (ω), is very similar to that for the individual lasers, which we can determine from the passive cavity losses. Instead, when the coupling is complex (ϕ > 0, Figure 1b), a difference in Im (ω) develops. Complex coupling arises from phase delay, obtainable through light propagation distances of the order of the wavelength, and can be implemented, for example, using a waveguide near the individual lasers.

**Figure 1:**
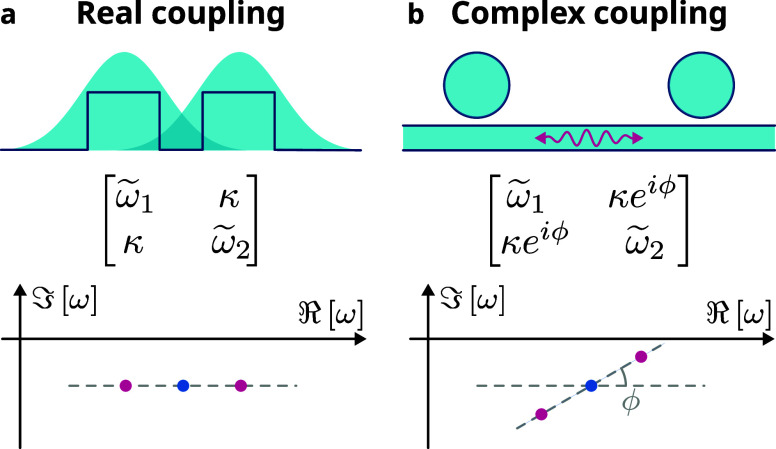
Complex coupling. (a) In coupled mode theory, coupling is typically considered real to account for the evanescent interaction between nearby particles. This results in the modes of the system splitting in frequency. (b) If the particles are far apart and coupled via radiation or waveguide modes, the coupling gains a phase and becomes complex. In general, this results in the coupled modes having not only different (real) frequencies but also different losses (imaginary frequencies).

When the two coupled lasers are pumped unequally, the threshold of each mode depends on both excitations.^21,22,38^ This dependence can be visualized as a threshold curve in the (*P*_1_, *P*_2_) plane as in Figure 2a, for ϕ = 0. The blue curve indicates the threshold of one of the modes (here, mode 1), which is now a wavy line (and would have been a straight line for uncoupled lasers). The linear gain increases with the pump *P*_*i*_, as indicated by the colormap.

**Figure 2:**
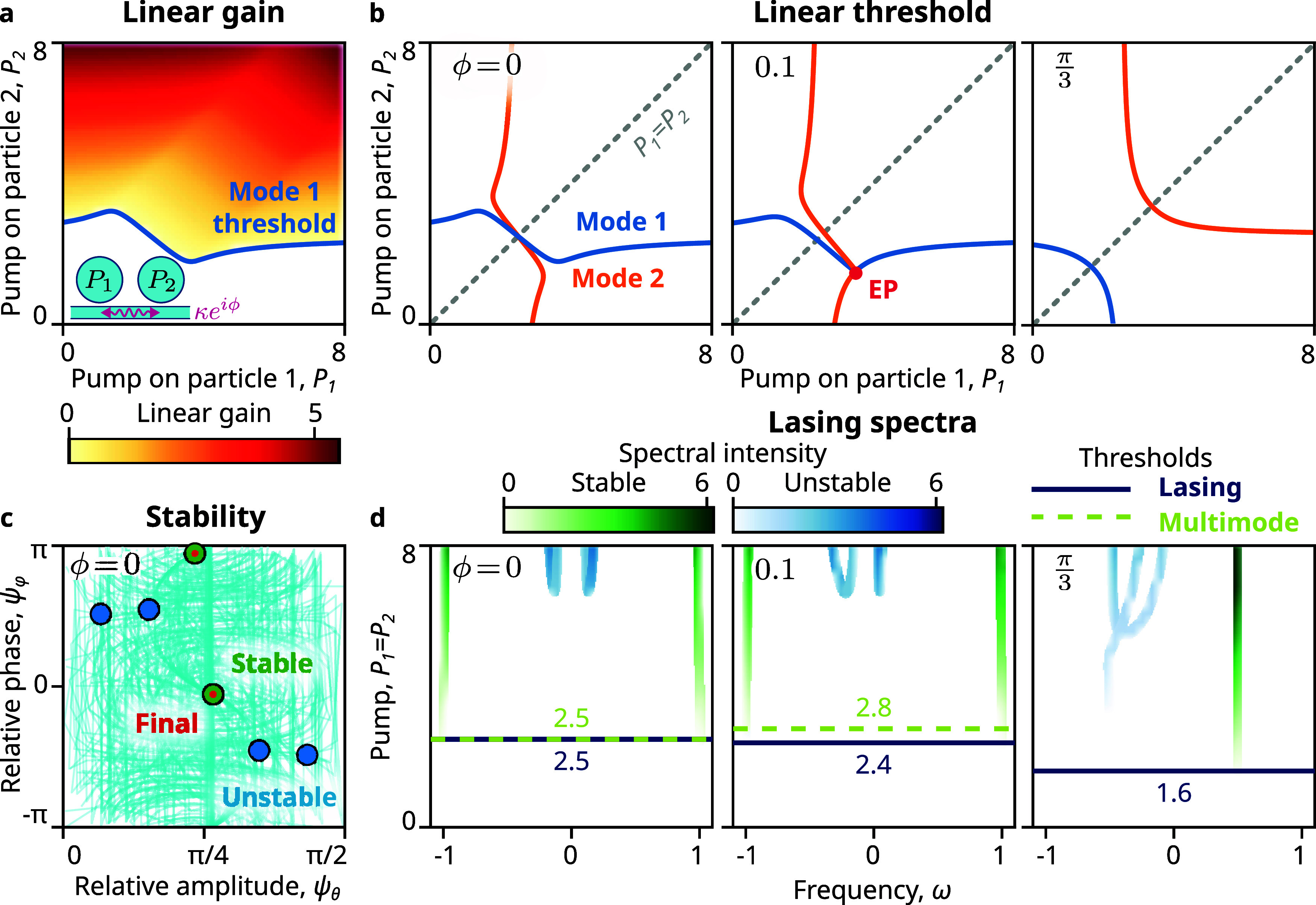
Single-mode lasing via complex coupling. (a) Two detuned lasers (ω_2_ – ω_1_ = 0.2, γ_1_ = γ_2_ = 2.5) interacting through complex coupling (*κe* ^*i*ϕ^, κ = 1) and pumped with (*P*_1_, *P*_2_). Under linear CMT with real coupling (ϕ = 0), described by the 2 × 2 matrix in Figure 1a, two coupled modes arise, which lase for pump values beyond the threshold curves (blue and orange), where their linear gain is positive. Linear gain of one of the modes is shown. (b) At ϕ = 0, the threshold curves cross symmetrically. On increasing ϕ, they become asymmetric and separate from each other at the exceptional point (ϕ = 0.1), yielding a large single-mode region at large delay (ϕ = π/3). (c) On evolving the system with random noise, the state trajectories (cyan) only evolve to the stable modes (green) identified by nonlinear CMT. (d) Under equal pump (*P*_1_ = *P*_2_), at ϕ = 0, two modes reach threshold simultaneously. On increasing ϕ, the two modes separate in threshold, with the second mode requiring an even higher pump to reach stability. For high values of ϕ, we obtain single-mode lasing operation, where only one mode is stable.

In the presence of complex coupling (ϕ > 0, Figure 2b), the threshold curves become asymmetric, specifically shifting their intersection away from the *P*_1_ = *P*_2_ line for increasing ϕ. Under an equal pump (gray dotted line), one mode reaches its threshold just before the other, enabling a limited range of single-mode operation. For a specific value of the coupling phase, here ϕ = 0.1, the eigenvectors of the two modes coalesce at an EP with anyonic PT symmetry. For ϕ > 0.1, the thresholds separate from each other with one curve becoming convex and the other, concave. Beyond this, one mode consistently reaches its threshold before the other, leading to a large range of single-mode operation.

However, above the threshold, linear CMT provides an incomplete picture with unphysical exponential growth of mode amplitudes with time. In real lasers, mode amplitudes are constrained by gain saturation, which we incorporate in nonlinear CMT^38,39^ (Supporting Section SIA). Nonlinearity leads to the emergence of more coupled modes.

We employ Jacobian stability analysis to assess the stability of these modes to identify the modes observable in experiments (Supporting Section SIA3). Under real coupling, only two modes exhibit stability, with equal intensities, as we expect from symmetry (Figure 2d). We confirm the stability of modes using time-domain simulations of the underlying coupled differential equations, starting from zero amplitude and adding random noise (Supporting Section SIA4). We consistently observe that the system state (cyan) converges to one of the stable modes (Figure 2c, green circles) and never to any unstable mode (blue circles), confirming that the modes assigned as stable are the experimentally observable ones.

Nonlinear CMT predicts an extended range of single-mode operation enabled by complex coupling compared to linear CMT. As the coupling phase ϕ increases, the lasing threshold decreases, and the second mode requires a higher pump intensity to attain stability than the linear onset (Figure 2d). At high values of ϕ, the second mode is never stable, resulting in a single stable lasing mode at all powers above the threshold. We attribute this observation to the significant separation between the threshold curves. Even with gain saturation, any mode arising from the higher threshold curve will retain enough gain for a mode from the lower curve to emerge and dominate it.

This single-mode operation can be achieved even when the two lasers are pumped unequally (Supporting Figure S1). Increasing ϕ narrows the parameter space for which a second stable mode exists, until it vanishes altogether. For a fixed total pump on the two nanolasers, going beyond the complex phase required for anyonic EP ensures stable single-mode lasing for all values of pump difference between them (Supporting Figure S2). Single-mode lasing is robust to small variations in total pump, differential pump, and phase delay, thus making it realistic (Supporting Figure S3). When the individual nanolasers are multimode, which limits the maximum pump that can be applied to them so as to not excite these higher modes, increasing the phase delay increases the maximum intensity of single-mode lasing supported by the coupled lasers (Supporting Figure S4). Further, single-mode lasing is attained at values of the coupling phase ϕ much lower than the studies of synchronization under dissipative coupling (ϕ = π/2).^35,36^

Introducing a high phase delay can thus effectively suppress multimode behavior in laser dimers, allowing for the sustained operation of a single stable lasing mode.

### Phase-Delayed Coupling in Realistic Systems

Realistic nanolasers are usually cylindrical,^40,41^ hexagonal,^38,42^ or spherical,^43^ while more complex 3D architectures are also starting to be investigated. We validate our model beyond the idealized CMT by investigating the coupling between the lowest-order vector spherical harmonic modes in a dimer of identical spheres based on Mie theory^44,45^ (Supporting Section SIB).

Phase-delayed coupling can be achieved in coupled sphere nanolasers by adding a physical distance between them. ϕ increases with the separation between the spheres, but at the expense of reducing the magnitude of the coupling strength κ as the fraction of the scattered light reaching the other sphere reduces.

The simulation in Figure 3a confirms that when two unpumped spheres are coupled, the coupled modes (purple line) have distinct values of real frequency and gain (imaginary part), indicating a complex effective coupling constant. As the pump is increased in both spheres equally, the gains of the modes increase, until eventually one mode reaches a lasing threshold on intersecting the real axis before the other, confirming our observations from CMT.

**Figure 3:**
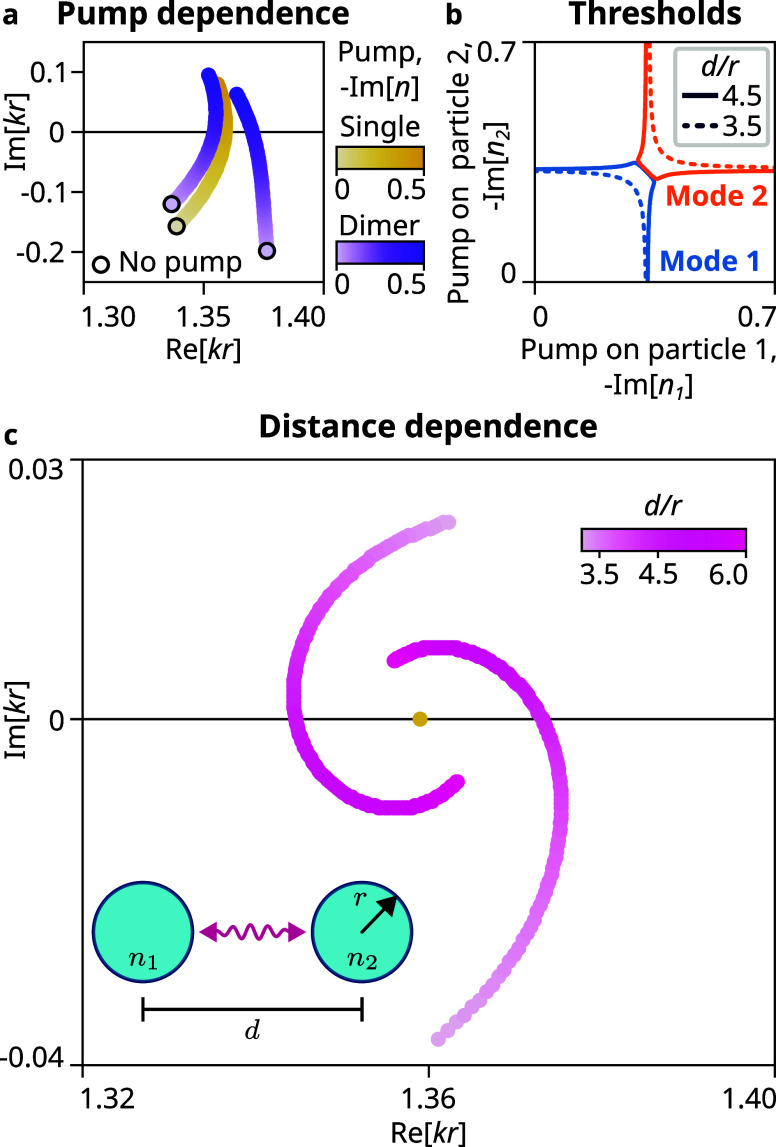
Complex coupling in a sphere dimer. (a) When two spheres of radius *r* separated by *d* are coupled, the mode of the sphere (brown) splits into two modes (purple) that differ both in frequency and gain. On pumping the spheres, one of the coupled modes reaches threshold before the other. (b) Threshold curves of the dimer vary in separation as the distance is varied, making one mode have more gain than the other. (c) Increasing the distance between the spheres changes both the amplitude and the phase of coupling, resulting in the coupled modes moving spirally around the single sphere mode.

This effect holds for all pump powers on the two spheres as shown in Figure 3b, where the threshold curve (Im [*kr*] = 0) is plotted for two intrasphere distances *d*/*r* = 4.5 (solid line) and *d*/*r* = 3.5 (dotted line), where *d* is the ratios between the distance and sphere radius. For *d*/*r* = 4.5, the threshold curves of the two modes intersect, resembling the prediction from CMT under real coupling; however, for *d*/*r* = 3.5, a noticeable gap emerges between the two threshold curves. This observation indicates that one mode requires significantly less pump power to reach the lasing threshold compared to the other, aligning with the prediction from CMT under highly complex coupling.

To demonstrate the tunability of the phase, we maintain a fixed and equal pump (corresponding to the threshold of a single sphere) and vary the distance *d*. The coupled modes of the system exhibit a spiral trajectory around the frequency of the mode of the single sphere (Figure 3c) due to two key factors. First, the continuous variation in the complex phase of the coupling makes the coupled modes encircle the single mode and separates them in the gain. Second, the magnitude of the coupling decreases as the spheres move apart, bringing the coupled mode frequencies closer to the single mode frequency. These results illustrate that manipulating the distance between the spheres in the dimer can effectively tune the phase of the coupling, in turn enabling us to optimize the lasing threshold and achieve single-mode operation (Supporting Figure S6). Due to the relatively slow decay of the coupling amplitude with distance (Supporting Figure S6a), care must be taken when analyzing arrays of more than two spheres. The typical approximation of nearest-neighbor interaction might be insufficient, and the phase-dependent correction from this approximation would be different for different eigenmodes of the collective system. Although we have considered the phase arising from scattering here for simplicity, more controllable and efficient coupling can be achieved by adding guided structures between the nanolasers, resulting in single-mode emission.^34,46^ Waveguiding would also allow tailoring of specific pairwise interactions, such as suppressing non-nearest-neighbor coupling. However, one has to then incorporate additional phase delays due to the coupling to the guided structures and material effects.

### Single-Mode Lasing in Large Arrays of Nanolasers

Single-mode operation due to phase-delayed coupling can be generalized to larger arrays of lasers. Here we calculate up to ten coupled lasers in a linear array, with nearest-neighbor interactions (Figure 4), using stochastic differential equations (Supporting Section SIA4). Phase-delayed coupling reduces the lasing threshold (green line), with an effect that increases when more and more lasers are coupled (Supporting Figure S5).

**Figure 4:**
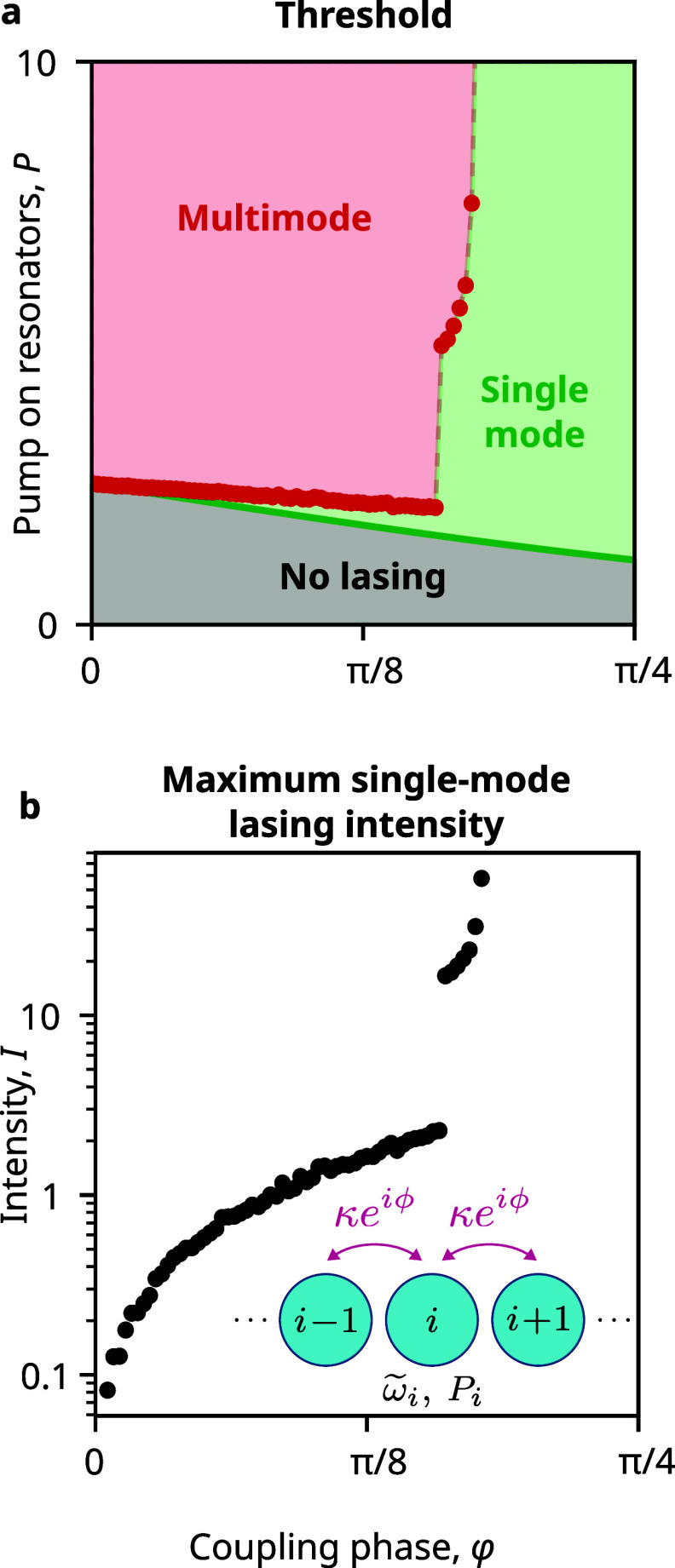
Single-mode lasing from large arrays. On increasing the coupling phase ϕ in a chain of *N* = 10 resonators with nearest-neighbor interactions that are pumped equally, the threshold of the fundamental mode reduces, and the range and intensity of single-mode operation increase. (a) Evolution of the lasing threshold of the fundamental mode (green line) and the highest pump power at which the array supports a single stable eigenmode (red points). Single-mode lasing is attained between these two limits. (b) Intensity of the stable eigenmode at the highest pump power that supports a single stable lasing mode.

The pump range permitting single-mode operation in the array also increases with the phase of coupling (Figure 4a). This is due to a combination of the same two factors seen previously in the dimer system: Increasing ϕ not only increases the linear gain difference between different modes but also makes the low-gain modes unstable and, thus, unattainable at low power. As some modes lose stability entirely at high values of complex phase, multimode onset increases drastically, allowing much higher power of single-mode lasing emission from the system. Unlike the dimer, the stochastic evolution of the 10-resonator system shows noneigen multifrequency solutions, but these are also suppressed with complex coupling. As a result, the maximum single-mode intensity, evaluated as the sum of squares of the amplitudes of the individual nanolasers in a frequency eigensolution, increases with complex coupling (Figure 4b).

These findings illustrate that phase-delayed coupling can achieve single-mode operation across a broad range of pump powers in large arrays. Although multiple modes and complex dynamical solutions exist in such arrays, phase-delayed coupling effectively suppresses them and allows a single mode with the lowest threshold to dominate the system.

## Conclusions

In conclusion, we have demonstrated the remarkable potential of phase-delayed coupling in achieving tunable single-mode lasing in nanolasers. Increasing the phase delay between coupled resonators suppresses both multimode eigensolutions and complex dynamical solutions in large arrays, sustaining a single stable lasing mode. The transition from multimode to single-mode lasing on increasing the phase delay is closely connected to the origin of anyonic PT symmetry, which is the critical point at which the coupled modes separate in the threshold space. Nonlinear CMT and stability analysis are crucial in describing coupled lasers, showing a more extended range of single-mode operation than predicted by linear CMT. Single-mode operation due to phase delay is robust to changes in system parameters, avoiding the strict requirements to match exceptional points. Our demonstration that phase-delayed coupling makes single-mode lasing possible in realistic systems and large arrays makes this a promising direction for the development of stable and high-power single-mode laser systems. Extending our analysis to incorporate physical effects such as resonator geometries, realistic semiconductor gain, and the phase of coupling through waveguides will provide a more comprehensive understanding of realistic single-mode lasers. This knowledge will aid in designing lasers for photonic chips for applications, such as optical communication, quantum information processing, and LIDAR.

## Supplementary Material



## Notes

Methods: nonlinear coupled mode theory (threshold modes, above-threshold solutions, stability analysis, time-domain simulations, and normalization); coupling in spherical dimers (theory, and coupling and threshold) (PDF)
