# Supplementary material for: Single-Mode Emission by Phase-Delayed Coupling Between Nanolasers
Source: ACS Photonics. 2025 May 5;12(5):2337–43. doi: 10.1021/acsphotonics.4c01230 (PMC12100714; doi:10.1021/acsphotonics.4c01230)
Supplement: Supplementary file 1 [file ph4c01230_si_001.pdf]

Supporting Information: Single-mode emission by phase-delayed coupling between nano-lasers

T. V. Raziman,[†,‡] Anna Fischer,[†,¶] Riccardo Nori,[†] Anthony Chan,[†] Wai Kit Ng,[†] Dhruv Saxena,[†] Ortwin Hess,[§] Korneel Molkens,[‖,⊥,#] Ivo Tanghe,[‖,⊥,#] Pieter Geiregat,[⊥,#] Dries Van Thourhout,[‖,#] Mauricio Barahona,[*,‡] and Riccardo Sapienza[*,†]

†*Blackett Laboratory, Department of Physics, Imperial College London, London SW7 2AZ, UK*

‡*Department of Mathematics, Imperial College London, London SW7 2AZ, UK*

¶*IBM Research Europe - Zürich, Rüschlikon 8803, Switzerland*

§*School of Physics and CRANN Institute, Trinity College Dublin, Dublin 2, Ireland*

‖*Photonics Research Group, Ghent University - Imec, 9052 Gent, Belgium*

⊥*Physics and Chemistry of Nanostructures Group, Department of Chemistry, Ghent University, 9000 Gent, Belgium*

#*Center for Nano- and Biophotonics, Ghent University, 9052 Gent, Belgium*

E-mail: m.barahona@imperial.ac.uk; r.sapienza@imperial.ac.uk

SI Methods

SIA Nonlinear Coupled Mode Theory

We model the coupled laser system using coupled mode theory (CMT) including gain saturation.[S1,S2] We had presented the two-laser system with real coupling in Ref. S2, which we generalise to N particles with complex coupling. Consider N single-mode resonators with resonant frequencies $\widetilde{\omega}_i = \omega_i - i\gamma_i$, coupled symmetrically by $\widetilde{\kappa}_{ij} = \widetilde{\kappa}_{ji} = \kappa_{ij} e^{\phi_{ij}}$ and pumped by P_i. The mode amplitude vector $\boldsymbol{\psi} = \left[\widetilde{\psi}_1, \ldots, \widetilde{\psi}_N\right]^T$ evolves according to the matrix equation,

$$\frac{d}{dt}\boldsymbol{\psi} = -i\boldsymbol{M}\boldsymbol{\psi}\,, \tag{S1}$$

where the diagonal elements of the $N \times N$ matrix $\boldsymbol{M}$ depend on the frequencies and the saturated pump,

$$M_{ii} = \widetilde{\omega}_i + \frac{iP_i}{1 + \left|\widetilde{\psi}_i\right|^2}\,, \tag{S2}$$

and the off-diagonal elements $M_{ij} = \widetilde{\kappa}_{ij}$ encode the coupling. We look for steady-state lasing solutions with real frequency ω given by

$$\boldsymbol{M}\boldsymbol{\psi} = \omega\boldsymbol{\psi}. \tag{S3}$$

This is a nonlinear eigenvalue problem as $\boldsymbol{M}$ depends on $\boldsymbol{\psi}$. Without loss of generality, we set the mean real frequency of the resonators to zero.

SIA1 Threshold modes

At threshold, $\widetilde{\psi}_i \to 0$, and we can replace $\boldsymbol{M}$ with the linear $\boldsymbol{M}^l$, with the same off-diagonal elements as $\boldsymbol{M}$ but diagonal elements $M^l_{ii} = \widetilde{\omega}_i + iP_i$. Finding the threshold pump $\boldsymbol{P} = [P_1, \ldots, P_N]^T$ becomes a linear eigenvalue problem

$$\boldsymbol{M}^l\boldsymbol{\psi} = \omega\boldsymbol{\psi}\,. \tag{S4}$$

Note that increasing all pump values by a constant, $P_i \to P_i + \Delta_P$, increases all eigenvalues of Eq. (S4) by $i\Delta_P$ without changing the eigenvectors. This observation provides a route to compute all threshold solutions. First, sweep over all $\boldsymbol{P}^0$ such that $\sum_i P^0_i = 0$ and compute the N eigenvalues $\widetilde{\omega}^j(\boldsymbol{P}^0)$ for $j = 1, \ldots, N$. Then, on modifying all the pumps, $P^0_i \to P^0_i - \mathrm{Im}[\widetilde{\omega}^j]$, mode j will attain threshold. The threshold is realistic if and only if all pump values satisfy $P_i \geq 0$. This is how the threshold curves are computed in Figure 2b.

SIA2 Above-threshold solutions

We find the self-consistent above-threshold solutions to the nonlinear Eq. (S3) from the linear threshold solutions. Let $\left(\boldsymbol{P}^{\mathrm{th}}, \omega^{\mathrm{th}}, \boldsymbol{\psi}^{\mathrm{th}}\right)$ be a threshold solution to Eq. (S4). Without loss of generality, we normalise $\boldsymbol{\psi}^{\mathrm{th}}$ such that $\sum_i \left|\widetilde{\psi}^{\mathrm{th}}_i\right|^2 = 1$ and assign the phase such that $\widetilde{\psi}^{\mathrm{th}}_1$ is

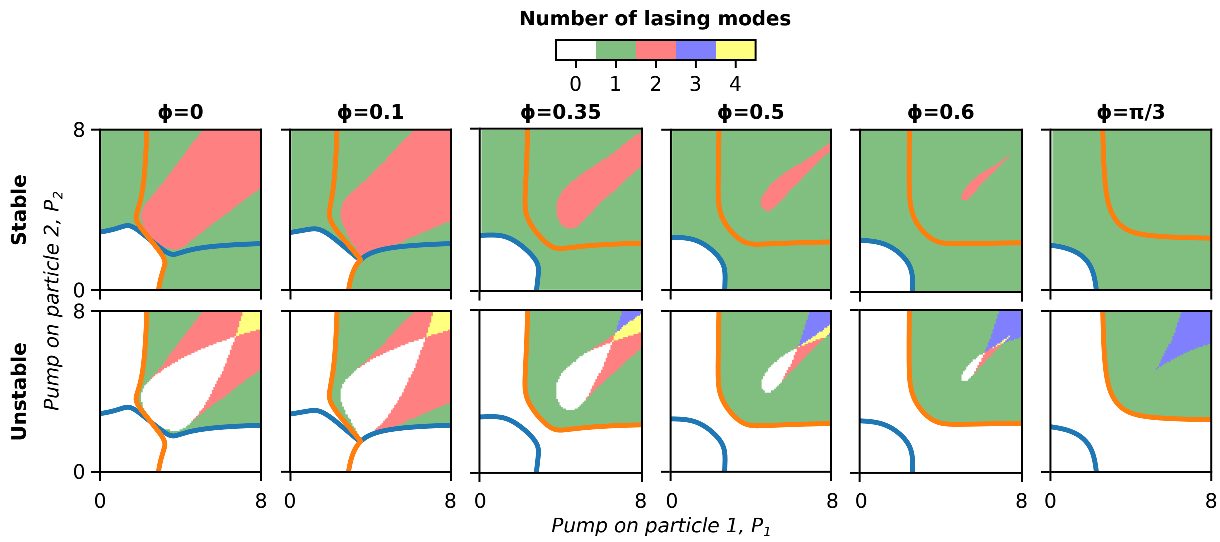

Figure S1: **Number of stable and unstable modes**. Under real coupling ($\phi = 0$), there are two stable modes when the two lasers are pumped approximately equally above threshold. Further unstable modes exist, with increasing number at high pump. As the coupling phase ϕ increases, the onset of the second stable mode is delayed, and eventually, all modes except the lowest-threshold mode become unstable.

real. For a mode amplitude $r \geq 0$, $\left(\boldsymbol{P}, \omega = \omega^{\text{th}}, \boldsymbol{\psi} = r\boldsymbol{\psi}^{\text{th}}\right)$ is a solution to Eq. (S3) where

$$P_i = P_i^{\text{th}} \left[1 + r^2 \left|\widetilde{\psi}_i^{\text{th}}\right|\right]^2 . \tag{S5}$$

As this transformation is invertible, it is easy to see that all above-threshold solutions can be found in this fashion from the threshold solutions. Effectively, the total pump in the system has been reduced to a threshold pump via gain clamping.[S3] The mode intensity, for instance plotted in Figure 2d, is evaluated as $I = r^2$.

SIA3 Stability analysis

We evaluate the stability of the above-threshold modes to small perturbations using Jacobian analysis. Near a steady-state solution $(\boldsymbol{P}, \omega, \boldsymbol{\psi})$, we have

$$\left(\frac{d}{dt} + i\omega\right) \boldsymbol{\psi} = -i \left(\boldsymbol{M} - \omega\right) \boldsymbol{\psi} = 0 \, . \tag{S6}$$

Separating $\boldsymbol{\psi}$ into real and imaginary parts, we can create a real mode amplitude vector $\boldsymbol{\Psi} = \left[\text{Re}(\widetilde{\psi}_1), \text{Im}(\widetilde{\psi}_1), \ldots, \text{Re}(\widetilde{\psi}_N), \text{Im}(\widetilde{\psi}_N)\right]^T$. We then transform $-i(\boldsymbol{M} - \omega)$ in Eq. (S6)

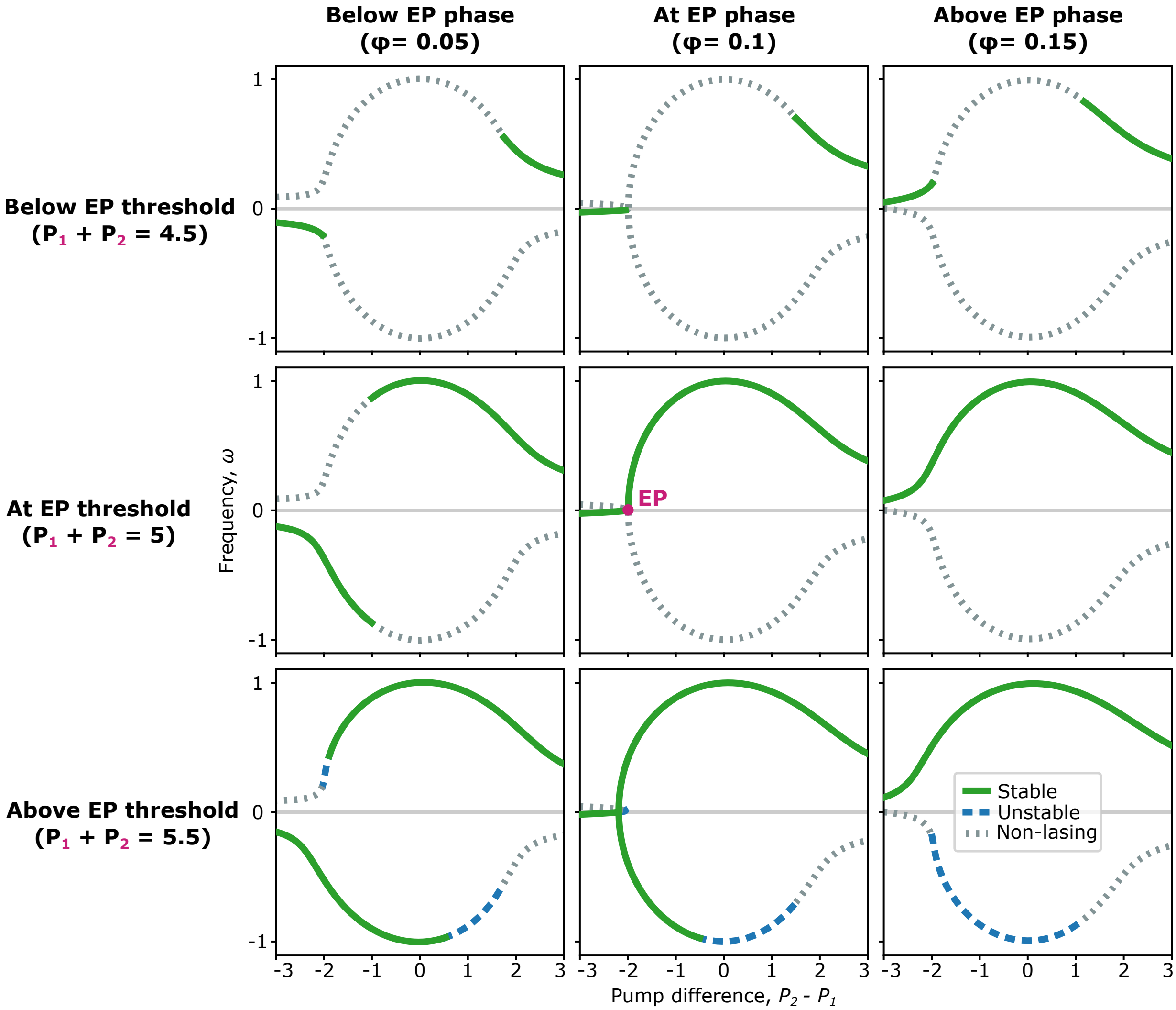

Figure S2: **Stable, unstable and non-lasing modes as a function of the difference between the pumps on the two nanolasers**. The columns vary the phase of coupling (left column: below the required phase for the anyonic EP, middle: at the anyonic EP, right: above the anyonic EP), and the rows vary the total pump (top: below the required total pump for the anyonic EP, middle: at the anyonic EP, bottom: above the anyonic EP). Increasing the pump results in more stable and unstable lasing modes. Increasing the complex phase results in the higher modes becoming unstable, resulting in single-mode lasing.

into a $2N \times 2N$ matrix $\boldsymbol{\mathcal{M}}$ that operates on $\boldsymbol{\Psi}$. The ith diagonal 2×2 block of $\boldsymbol{\mathcal{M}}$ is

$$\boldsymbol{\mathcal{M}}_{ii}^{2\times 2} = \begin{bmatrix} -\gamma_i + \dfrac{P_i}{1 + \left|\widetilde{\psi}_i\right|^2} & \omega_i - \omega \\ -\omega_i + \omega & -\gamma_i + \dfrac{P_i}{1 + \left|\widetilde{\psi}_i\right|^2} \end{bmatrix}, \tag{S7}$$

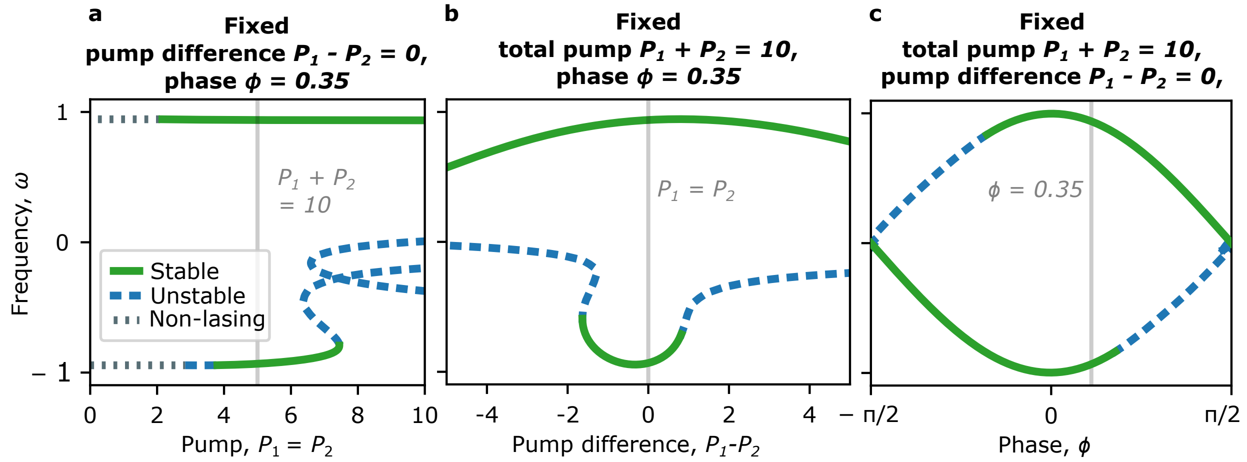

Figure S3: **Change in stable, unstable and non-lasing modes near** $P_1 = P_2 = 5, \phi = 0.35$ **under variations** of a) total pump, b) pump difference, and c) phase of coupling.

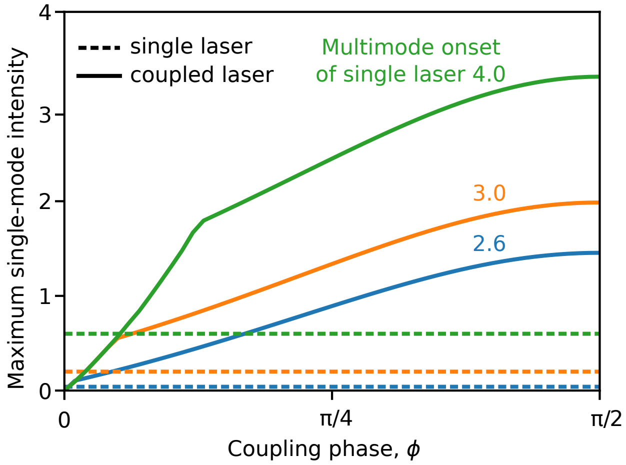

Figure S4: **Maximum single-mode lasing intensity in a dimer of multimode nanolasers**. Suppose that the individual nanolasers in Fig. 2, with a fundamental threshold of 2.5, support another mode at a higher pump (blue: 2.6, orange: 3.0, or green: 4.0) that does not take part in coupling, so that the laser has to be pumped below this level. Maximum single-mode intensity in the individual nanolaser is shown by dashed lines. In the coupled dimer under equal pumping, the maximum single-mode intensity is zero for $\phi = 0$ due to the existence of two modes with identical gains. As the coupling phase increases, the maximum single-mode intensity also increases. Two distinct regimes are visible: At low ϕ, the maximum single-mode intensity rises rapidly due to the increase in stable multimode threshold seen in Fig. 2d. Once the stable multimode threshold goes above the onset of the higher-order mode of the individual nanolasers, the pump can no longer be increased - but the maximum single-mode intensity continues to increase due to the lowering of the threshold. In all cases, complex coupling permits higher single-mode lasing intensity than possible from a single nanolaser.

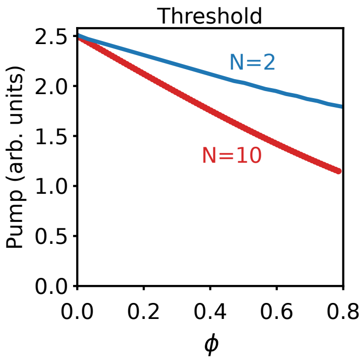

Figure S5: **Lasing threshold of the dimer and a chain of $N = 10$ particles**. The thresholds of the two systems are the same under real coupling ($\phi = 0$). As the coupling phase ϕ increases, the threshold of the larger array drops faster.

and the ij^{th} off-diagonal 2×2 block is

$$\boldsymbol{\mathcal{M}}_{ij}^{2\times 2} = \begin{bmatrix} \mathrm{Im}(\widetilde{\kappa}_{ij}) & \mathrm{Re}(\widetilde{\kappa}_{ij}) \\ -\mathrm{Re}(\widetilde{\kappa}_{ij}) & \mathrm{Im}(\widetilde{\kappa}_{ij}) \end{bmatrix} . \tag{S8}$$

For the mode in the steady state, $\boldsymbol{\mathcal{M}}\boldsymbol{\Psi} = 0$. The mode is stable if small perturbations decay with time, which can be checked by evaluating the eigenvalues of the Jacobian matrix. The state is stable if and only if all eigenvalues have a negative real part (except for one which will be zero, corresponding to phase invariance). This is how modes are assigned stable or unstable in Figures 2d and S1.

SIA4 Time-domain simulations

We perform time-domain simulations to confirm the assignment of stable modes in Figure 2c. We start the system at $\boldsymbol{\psi} = 0$ and evolve it based on Eq. (S1). We use the forward Euler method with a time step of $0.01/|\kappa|$, and add random noise with strength 10^{-5} at each time step. At the end of the simulation (1000 time steps), we remove the global phase from the system by enforcing $\widetilde{\psi}_1$ to be real.

We also use time-domain simulations to identify multimode onset in Figure 4. We follow this instead of the analytical procedure in section SIA2 since it cannot deal with noneigen solutions. For each value of ϕ, we perform an exponential + binary search to find the upper limit for single-mode lasing. We start at a pump 0.01 above the lasing threshold and increment this difference in multiples of 1.5. 20 simulations are performed at the pump for 10^5 time steps ($\mathrm{dt} = 0.01$), and the addition of random noise is stopped after 1000 time steps. Lasing is considered stable if and only if all simulations result in identical (to a tolerance of 10^{-5}) solutions which are eigensolutions. A solution is considered an eigensolution if it is invariant with time up to the tolerance, once global phase is removed. After this exponential search concludes, a binary search is performed between the lower and upper limits in the same way to find the single-mode threshold to an accuracy of 0.01.

SIA5 Normalisation

All the theory and calculations in the manuscript have been normalised to work with non-dimensional quantities. We now provide a recipe to convert these non-dimensionalised values into experimentally relevant quantities.

First, scale the amplitude $\widetilde{\psi}$ of a single uncoupled nanolaser such that $I = \left|\widetilde{\psi}\right|^2$ gives the output power of the nanolaser. Eq S1 governing the evolution of the amplitude can then be written as,

$$\frac{d}{dt}\widetilde{\psi} = \left[-i\omega - \gamma\left(1 - \frac{P/P^{th}}{1 + |\widetilde{\psi}|^2/I_0}\right)\right]\widetilde{\psi}\,, \tag{S9}$$

where P^{th} is the threshold pump the nanolaser requires to lase, and I_0 is the intensity normalisation factor. The steady-state eigensolutions then provide the output lasing power,

$$I = I_0\left(\frac{P}{P^{th}} - 1\right). \tag{S10}$$

The intensity-normalisation factor I_0 can thus be found from the slope of the light-in-light-out (LL) curve of the laser. The remaining quantities (ω, γ, κ) can be expressed in frequency units following the treatment in Ref. S2.

SIB Coupling in spherical dimers

We use Mie theory[S4] to compute the coupled modes of a dimer of spheres in vacuum ($n_b = 1$). For a sphere with radius r, we model single-mode operation by approximating that only the lowest-order electric vector spherical harmonic mode $\mathbf{N}_{1,0}$ is present in the two spheres. For an individual sphere with refractive index n_1, from the continuity of the electric and magnetic fields at the surface, we obtain the condition for the existence of a mode to be the vanishing of the matrix determinant

$$\begin{vmatrix} \frac{1}{\rho_1}\left[\rho_1 j(\rho_1)\right]' & \frac{1}{\rho}\left[\rho h^{(1)}(\rho)\right]' \\ \rho_1 j(\rho_1) & \rho h^{(1)}(\rho) \end{vmatrix} = 0\,, \tag{S11}$$

where k is the complex wavenumber, $\rho = kr$, $\rho_1 = kn_1r$, j is the spherical bessel function of the first kind, and $h^{(1)}$ is the spherical hankel function of the first kind. As we limit ourselves to the $\mathbf{N}_{1,0}$ mode, both spherical functions are of order 1 and the subscript (j_1, h_1) is omitted. As the physics is size invariant, we set $r = 1$ and search in the complex plane for a value of k that satisfies Eq (S11) to find the mode kr of a single sphere. We set the unpumped refractive index of the sphere to be 3.2, and set the imaginary part of the index to increase linearly with the pump. This is how the evolution of the mode of the single sphere is plotted in Figure 3a.

We now consider two identical spheres with centres located at $\pm d/2\hat{z}$ so that their centre-to-centre separation is d. Their refractive indices are n_1 and n_2, both having unpumped values of 3.2 and negative imaginary parts increasing linearly with the respective pumps. We compute the amplitude of the mode of one sphere at the surface of the other using the

addition theorem for vector spherical harmonics.[S5] The condition for the dimer modes is

$$\begin{vmatrix} \frac{1}{\rho_1}\left[\rho_1 j(\rho_1)\right]' & \frac{1}{\rho}\left[\rho h^{(1)}(\rho)\right]' & 0 & \frac{A}{\rho}\left[\rho j(\rho)\right]' \\ \rho_1 j(\rho_1) & \rho h^{(1)}(\rho) & 0 & A\rho j(\rho) \\ 0 & \frac{A}{\rho}\left[\rho j(\rho)\right]' & \frac{1}{\rho_2}\left[\rho_2 j(\rho_2)\right]' & \frac{1}{\rho}\left[\rho h^{(1)}(\rho)\right]' \\ 0 & A\rho j(\rho) & \rho_2 j(\rho_2) & \rho h^{(1)}(\rho) \end{vmatrix} = 0\,, \tag{S12}$$

where $\rho_2 = kn_2r$. The coupling term A comes from the addition theorem for vector spherical harmonics, and is given by

$$A = \frac{3}{kd}h^{(1)}(kd)\,. \tag{S13}$$

We find the modes of the dimer in Figure 3a,c by finding the values of k in the complex plane satisfying Eq. (S12).

We compute the threshold curves for the coupled modes of the dimer in Figure 3b by sweeping the pump difference between the two spheres from -0.4 to 0.4. This is analogous to the $\mathbf{P}^0$ sweep in section SIA1. For each value of the pump difference, we increase the pump on the two spheres until the modes reach the real k axis.

SIB1 Coupling and threshold

The splitting in the real and imaginary parts of the modes in Fig. 3c allow us calculate the magnitude and phase of coupling as a function of separation between the spheres (Fig S6 a,b). The asymmetric splitting of the modes due to coupling and the complexity of adding nonlinearity to Mie theory make it difficult to perform the complete analytical calculation of multimode thresholds in the dimer. We therefore approximate the system by nonlinear CMT, by repeating the treatment in section SIA for a dimer with γ given by the loss of the single sphere, and κ found from the mode splitting (Fig S6 a,b). Fig. S6c presents the lasing and multimode thresholds found by this procedure. The multimode threshold is the same as the lasing threshold when the complex phase $\phi = 0$, and increases with increasing ϕ. Eventually, the multimode threshold diverges, permitting single-mode lasing at high powers.

References

(S1) Benzaouia, M.; Stone, A. D.; Johnson, S. G. Nonlinear exceptional-point lasing with ab initio Maxwell–Bloch theory. *APL Photonics* **2022**, *7*, 121303.

(S2) Fischer, A.; Raziman, T. V.; Ng, W. K.; Clarysse, J.; Saxena, D.; Dranczewski, J.; Vezzoli, S.; Schmid, H.; Moselund, K.; Sapienza, R. Controlling lasing around exceptional points in coupled nanolasers. *npj Nanophotonics* **2024**, *1*, 6.

(S3) Santos, E. P.; Silva, R. F.; Silva, J. F.; Maciel, C. V. T.; Luz, D. F.; Lima, E. D.; Maia, L. J. Q.; Lima, B. C.; Moura, A. L. Gain clamping in random lasers. *Laser Phys. Lett.* **2021**, *18*, 125002.

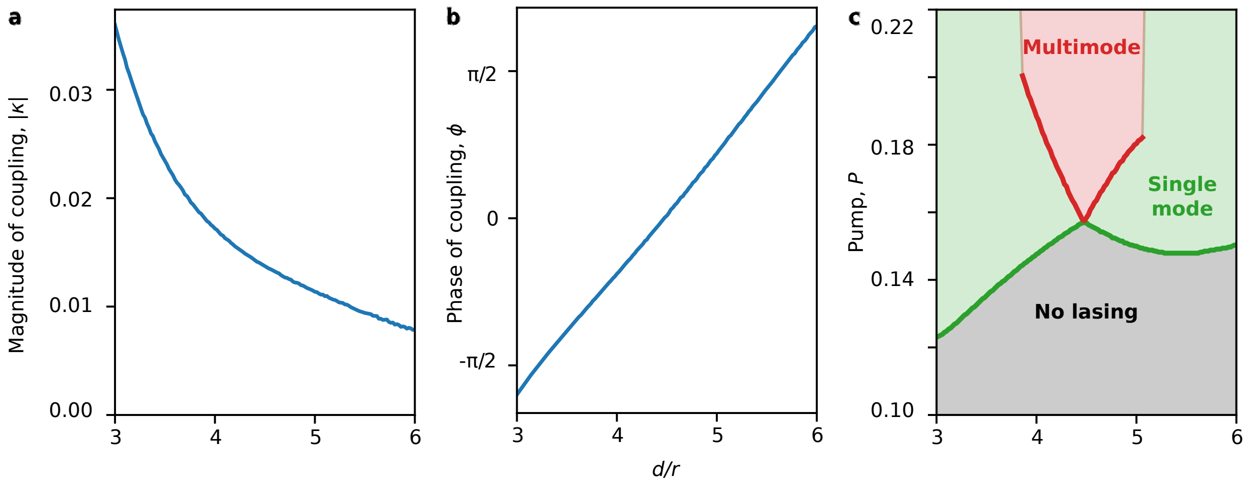

Figure S6: **Distance dependence of coupling and threshold in the spherical dimer**. a) Magnitude, and b) phase of the coupling. c) Lasing and multimode thresholds found by approximating the pump-dependence of the dimer with CMT.

(S4) Bohren, C. F.; Huffman, D. R. *Absorption and Scattering of Light by Small Particles*; John Wiley & Sons, Ltd, 1998; Chapter 4, pp 82–129.

(S5) Chew, W. C. *Waves and Fields in Inhomogeneous Media*; IEEE Press, 1998; Chapter D, pp 591–597.
